# Scheduling optimisation of alcohol test sites

**DOI:** 10.1038/s41598-024-63026-7

**Published:** 2024-05-28

**Authors:** Hongjun Yu, Emily Moylan, Mike Bambach, David Levinson, Mohsen Ramezani

**Affiliations:** School of Civil Engineering, The University of Sydney, Sydney, Australia

**Keywords:** Engineering, Civil engineering

## Abstract

Drink driving is an infamous factor in road crashes and fatalities. Alcohol testing is a major countermeasure, and random breath tests (RBTs) deter tested drivers and passersby (observers who are not tested). We propose a genetic algorithm (GA)-based RBT scheduling optimisation method to achieve maximal deterrence of drink driving. The RBT schedule denotes the daily plan of where, when, and for how long tests should occur in the road network. The test results (positive and negative) and observing drivers are considered in the fitness function. The limited testing resource capacity is modeled by a number of constraints that consider the total duration of tests, the minimum and maximum duration of a single test site, and the total number of test sites during the day. Clustering of the alcohol-related crash data is used to estimate the matrix for drink driving on the scheduled day. The crash data and traffic flow data from Victoria, Australia are analysed and used to describe sober/drink driving. A detailed synthetic example is developed and a significant improvement with 150% more positive results and 59% more overall tests is observed using the proposed scheduling optimisation method.

## Introduction

Drink driving is a major cause of fatal crashes. As a countermeasure, random breath tests (RBTs) were implemented decades ago on a global scale. The program has been effective in deterring potential drink driving and increasing overall road safety in many countries^1^.

To improve RBT performance, some testing operations specify target areas. This was investigated in^2^, and it was found in Victoria, Australia that there were more positive breath test results in targeted areas with high alcohol prevalence in crashes/drivers. Better discretion to stop and test target drivers tends to have higher successful detection rates^3^. A traffic enforcement resource allocation model in^4^ and^5^ was developed to assist police in the deterrence of drink driving. In a study in South Australia^6^, it was reported that RBTs that adopted the targeted-RBT approach captured 29 drink drivers per one thousand breath tests performed, in comparison with 5.7 drivers otherwise. We seek to optimise the locations and times of RBT sites to facilitate more tests and capture more positive test results to achieve sustained safety performance.

Drivers infer the likelihood of apprehension by the observed level of police enforcement intensity. In 2016 and 2020, the New Zealand Transport Agency (NZTA) conducted two rounds of a Public Attitudes to Road Safety Survey on drivers’ expectations of alcohol tests^7^ and^8^. In both years, the survey results indicated that drivers had correctly recognised 10pm-12am as the time period with the highest likelihood of encountering RBTs. It was found by^9^ and^6^ that in the early evening (before 6:00 pm), if potential drink drivers observed roadside breath test operations on the way to drinking venues, then they would re-evaluate whether to drink and drive. Strategic temporal allocation of RBT sites could change drivers’ attitudes towards drink driving, increase positive test rates, and enforce the general deterrence of drink driving.

Locations and times of RBT sites may be scheduled on a daily basis. A meta-analysis in^10^ focused on roadside breath test locations and their impacts on crashes. It was found that the largest crash reductions were found in the first 3-6 months after the establishment of a new test site. The randomness of RBT sites weakens the ‘grapevine effect’ and strengthens the deterrence by observations of test sites^6^. In rural areas, unexpected RBTs can achieve better efficiency and sustain longer deterrence with limited police resources^11^ and^12^. The study used a control group and reached a similar conclusion from test sites at the best-fixed locations to the randomly alternating sites across potential checkpoints. The National Highway Traffic Safety Administration (NHTSA) in the United States proposed alternative enforcement with flexible checkpoints, sometimes referred to as ‘phantom checkpoints’ or ‘mock’ sites, to supplement traditional test sites^13^.

An effective measure to reduce alcohol-related road traumas is to increase testing capacity^12^. The available police resources define the overall testing capacity, and the duration of each testing site is vital to anticipate the number of tests. In a study regarding RBTs^1^, the duration was defined as the time elapsed between the first and the last breath test conducted at an RBT site. With a larger testing capacity, an RBT site can obtain more test results per unit of time, which means more possible positive test results. In the proposed optimization method, we will use a number of inequalities to accurately describe these operational constraints and find the optimal RBT schedules within the testing capacity.

We present a Genetic Algorithm (GA)-based approach for the optimisation of test scheduling under limited resources. The approach mathematically describes the testing schedules as a constrained optimisation program given the restrictions on limited working capacity and testing force (police personnel). The objective function of the optimisation program is to maximise the combination of positive test results and the overall number of tests. The contributions of our research are summarised as follows:

- Mathematically formulate the problem of roadside test scheduling while considering constraints of limited police resources;
- Define a fitness function that takes measured traffic flow and RBT test results into account;
- Cluster alcohol-related crash data by date types and adjust the RBT allocations to weekdays, weekends, and public holidays, which adds randomness to the locations and times of test sites;
- Propose a simulation-based procedure and design a GA optimisation scheme that achieve maximal general deterrence.

The remainder of the paper is organised as follows. We start with the problem statement in Section "Problem statement". An outline of the goals, approaches, and assumptions in this study will be given. Then, we present the definitions of testing schedules and the fitness function in Section "Problem formulation". We propose the GA-based optimisation approach and present clustering of alcohol-related crashes in Section "Optimisation method". Data analysis, numerical experiments setup, and simulation results are presented in Section "Data and numerical experiments". Limitations of the proposed approaches and future work are discussed in Section "Limitations and future work". The research is summarized in Section "Summary".

## Problem statement

One goal of roadside RBT is to maximise general deterrence to drivers such that they are dissuaded from drink driving in the future. Such deterrence is achieved by delivering alcohol tests to all drivers (sober drivers who could potentially drink drive in the future, and drink drivers who could potentially cause road casualties). There are several different types of deterrence against drink driving. Specifically, direct contact with random breath testing has the strongest deterrent impact on drink driving^3^. Delivering more tests generates higher deterrence, while indirect contact, such as delivering tests in a high-traffic area, has less impact^6^. We combine these circumstances and use the number of tests and observing drivers to quantify the effectiveness (general deterrence) of a roadside testing schedule. The number of positive test results is used as an additional item in the measurement of the general deterrence scores. Intuitively, more positive test results lead to a higher likelihood of drink driving being captured, which helps discourage recidivism and mitigate the potential casualty caused by drink driving. The higher the deterrence score is, the larger the deterrence the schedule is expected to deliver.

The location and the start and finish times characterise a test site. On a given day (24 h), the set of all testing sites (location and time) constitutes the RBT schedule. The attributes of a testing schedule include the total number of test sites, the duration of individual test sites, and the total testing duration. Testing schedules are restricted due to limited resources. A schedule consists of a limited number of test sites because there are limited trained police personnel and equipment; the duration of a test site is lower and upper bounded because the police can only work on reasonable rosters; and due to the overall limited budget, there is an upper bound on the maximum total duration of all tests. A testing schedule is eligible only if it satisfies all the constraints, and the schedule is optimal if it achieves the maximal fitness value. We define the fitness function based on the number of tests and observing-but-untested (passerby) drivers. We propose a GA-based approach to optimise the testing schedules.

To fully capture the effect of RBT schedules on positive and negative test results and general deterrence, we should model drink and sober driving in the network. The spatial and temporal characteristics of vehicle-based travel may differ between drink and sober drivers. The likelihood of drivers drinking and driving varies based on the time of day and location. We use alcohol-related crash data to reconstruct the temporal/spatial distributions of drink driving and use traffic flow data to find out the distribution of sober driving.

Police recordings of RBTs and traffic flow sensors (e.g., loop detectors) provide estimates of drink drivers and counts of passing vehicles on different links. However, due to confidentiality around RBT results and the scarcity of traffic flow measurement sensors (e.g., at the state level), drink driving and traffic flow data are only partially available. We estimate traffic flow on roads where there are no traffic flow sensors. We also infer the occurrence of drink driving on roads with no police RBT records solely based on alcohol-related crash data. After the temporal/spatial distributions of drink and sober driving are obtained, we use them to optimise schedules of roadside alcohol tests and generate simulated drink driving instances.

**Figure 1:**
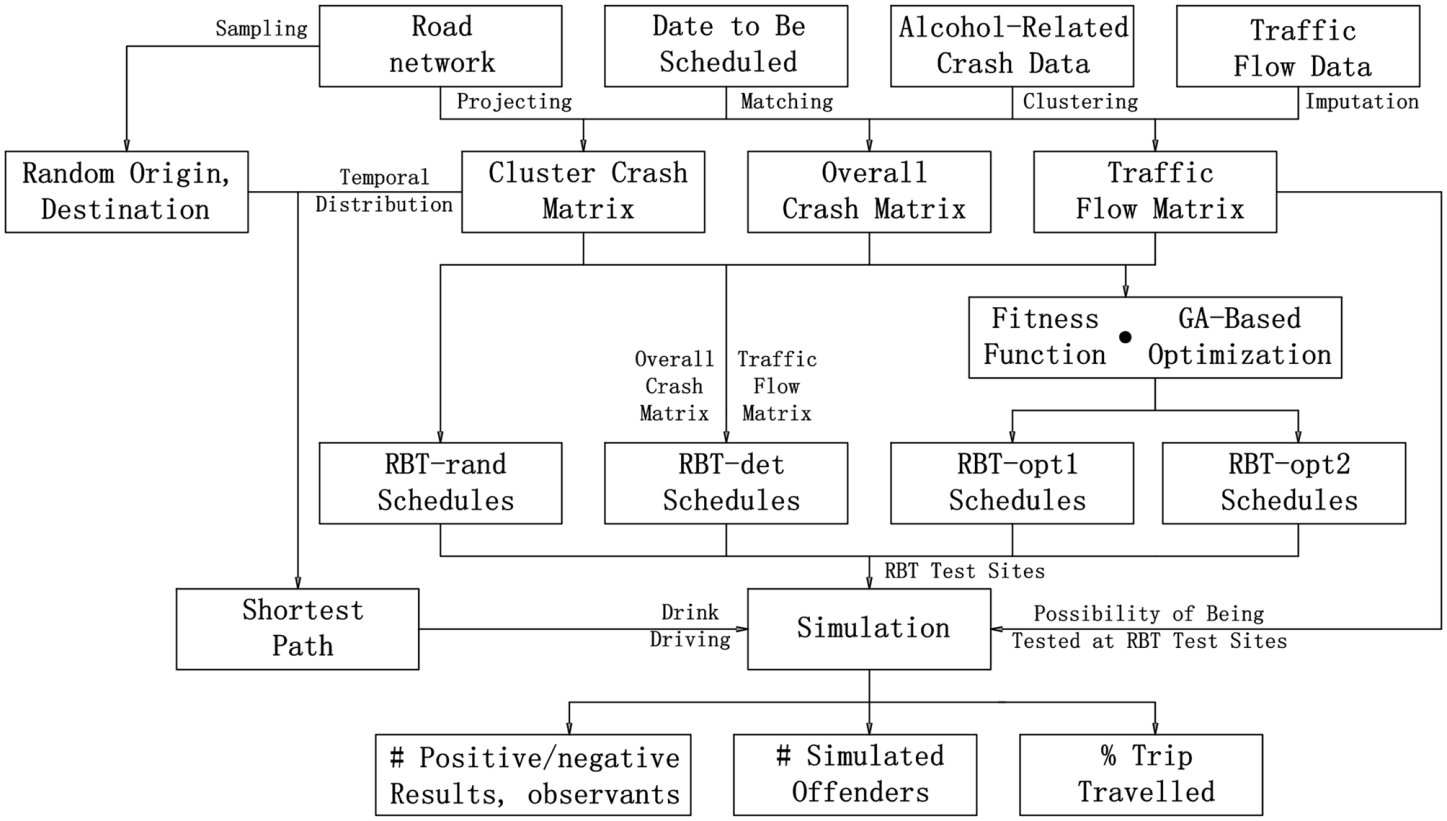
Flowchart for test scheduling optimisation.

Figure 1 illustrates the flowchart of the test scheduling optimisation process. First, to take advantage of available alcohol-related crashes through generalisation, we partition the alcohol-related crash data into a number of clusters using the K-medoids clustering method^14^. Attributes of the data points include the time of each crash and the total number of daily crashes on each road segment. The days are labelled with weekdays, weekends, and public holidays. A single crash is regarded as an instance, and the severity is not considered. Then, alcohol-related crashes are projected onto the road network by latitude and longitude. The crashes are categorised into $t^*$ time intervals per road segment to form the overall crash matrix (without loss of generality, we assume $t^*=96$, i.e., a day is divided into 96 15-min intervals).

We also project the latitude and longitude of the available traffic flow data to the road network. We consider the number of vehicles, the time intervals (15-min), and the locations (the road segments) from traffic flow sensors to complement flows on roads with no traffic flow sensors (see Section "Traffic flow imputation").

We use four types of RBT schedules for benchmarking:

- The random RBT schedules (RBT-rand) are generated with random start/finish times and random locations sampled from the road network.
- The deterministic RBT schedules (RBT-det) are obtained using the cluster crash matrix and traffic flow matrix by a deterministic algorithm. The aim of this benchmark is to schedule RBTs based on historical alcohol-related crashes. Its performance will provide the baseline for the evaluation of the optimised RBT schedules.
- Optimised RBT-opt1 schedules are obtained by the GA-based approach using the *cluster* crash matrix and the traffic flow matrix. In the process, we combine the cluster crash matrix and the traffic flow matrix to calculate the fitness values of the RBT-opt1 schedules, which are used by GA to optimise the testing schedules.
- Optimised RBT-opt2 schedules are obtained by the GA-based approach using the *overall* crash matrix and the traffic flow matrix. In the process, we combine the overall crash matrix and the traffic flow matrix to calculate the fitness values of the RBT-opt2 schedules, which are used by GA to optimise the testing schedules. This benchmark is considered to evaluate the effectiveness of crash data clustering on the performance of RBT schedule optimisation.

More details are presented in Sections "Benchmark methods" and "RBT performance results".

We use simulations to verify the performances of RBT schedules. The simulated drink drivers are generated between random origins and destinations using the shortest travel time paths and starting at times drawn from cluster temporal distributions. The likelihood of being tested at RBT test sites is calculated based on the traffic flow values. The simulation outputs include the numbers of positive and negative test results, and the percentage of trip travelled for each identified simulated offender.

## Problem formulation

This section presents the mathematical descriptions for the RBT schedules, constraints, and the fitness function. The definitions and inequalities will be used for the optimisation and analysis of RBT scheduling performance.

### Test schedule formulation

We consider the RBT to be scheduled daily. In theory, the start and finish times of test sites are continuous; in practice, the times may be rounded to the quarter hour. We divide the 24 h in a day into $t^*$ intervals of $1440/t^*$ min. Thus, the *t*-th interval is from *hour* : *minute* to $hour:minute+15$, where *hour* and *minute* are the quotient and remainder of $(t-1)/4$.

The value of the testing state on any road segment is regarded as binary (1 or 0) where 1 means that testing is scheduled on the road segment and 0 means no testing is scheduled on the road segment. The state value stays 1 for the duration of the testing on the road segment. In practice, the location of a test site on a road segment may be random. For simplicity, we assume that the locations are always at the midpoint of the road segment. Moreover, we define the testing states of any road segment by a vector of $t^*$ elements. The testing schedule is denoted as the matrix of all the state vectors for all the road segments on the day.

We use binary matrix $S(\kappa )$ of testing states $s_{i,t}(\kappa )\in \{0,1\}$ in (1) to denote the testing schedule on day $\kappa$:

1 $$\begin{aligned} S(\kappa )=\left[ \begin{array}{c} S_1(\kappa )\\ \vdots \\ S_i(\kappa )\\ \vdots \end{array}\right] =\left[ \begin{array}{cccc} s_{1,1}(\kappa )&{}s_{1,2}(\kappa )&{}\dots &{}s_{1,t^*}(\kappa )\\ \vdots &{}\vdots &{}\ddots &{}\vdots \\ s_{i,1}(\kappa )&{}s_{i,2}(\kappa )&{}\dots &{}s_{i,t^*}(\kappa )\\ \vdots &{}\vdots &{}\ddots &{}\vdots \end{array}\right] , \end{aligned}$$

where $S_i(\kappa )$ is the testing state vector of road segment *i* on day $\kappa$; $s_{i,t}(\kappa )$ is the testing state value for road segment *i* during *t*-th interval on day $\kappa$.

The testing schedules can also be defined by the test sites, which are described by the start and finish times and the location. We can use these attributes of test sites to define the testing schedule ${\tilde{S}}(\kappa )$ below.

2 $$\begin{aligned} {\tilde{S}}(\kappa )= & {} \Bigl \{\{{\bar{t}}_1,{\underline{t}}_1,i_1\},\dots ,\{{\bar{t}}_N,{\underline{t}}_N,i_N\}\Bigl \}, \end{aligned}$$

3 $$\begin{aligned}{} & {} {\bar{t}}_1<{\underline{t}}_1,\dots ,{\bar{t}}_N<{\underline{t}}_N, \end{aligned}$$

where there are *N* test sites in total; test site $g_w(\kappa )$, $1 \le w \le N$, starts from ${\bar{t}}_w$ and lasts till ${\underline{t}}_w$ on road segment $i_w$; $1\le {\bar{t}}_1,\dots ,{\bar{t}}_N\le t^*$ and $1\le {\underline{t}}_1,\dots ,{\underline{t}}_N\le t^*$.

Both $S(\kappa )$ and ${\tilde{S}}(\kappa )$ can be used to describe the testing schedule on day $\kappa$. Note that ${\tilde{S}}(\kappa )$ and $S(\kappa )$ are mutually convertible. $S(\kappa )$ is useful in deriving the number of positive results, the total number of tests and observing drivers, and analysing the performance of testing schedules. ${\tilde{S}}(\kappa )$ is expressed as a sequence, which is suitable for GA-based optimisation. The variable ${\tilde{S}}(\kappa )$ is used in Algorithm 1 and $S(\kappa )$ is used to calculate fitness values in Section "Fitness function".

Police resources and funding are usually limited. In addition, the duration of a test site is lower- and upper-bounded as police normally work on predefined rosters. The maximum total duration of testing in one day is also limited. The testing schedule $S(\kappa )$ or ${\tilde{S}}(\kappa )$ is subject to the aforementioned constraints. We use the inequalities below to describe the constraints.

4a $$\begin{aligned}{} & {} N\le N_{\max }, \end{aligned}$$

4b $$\begin{aligned}{} & {} t_{\min }\le {\underline{t}}_w-{\bar{t}}_w\le t_{\max }, \quad \forall g_w(\kappa ),\end{aligned}$$

4c $$\begin{aligned}{} & {} \underset{w=1}{\overset{N}{\sum }}({\underline{t}}_w-{\bar{t}}_w)\le \Delta , \end{aligned}$$

where there are maximum $N_{\max }$ test sites in total during a day (4a); testing duration at any test site is not shorter than $t_{\min }$ hours and not longer than $t_{\max }$ hours (4b); and the total duration of all testings is no longer than $\Delta$ hours (4c). Note that the inequalities in (4a–4c) are used in GA-based optimisation to obtain the optimised testing schedule.

### Traffic flow imputation

The total number of tests is a crucial part of RBT and consequent general deterrence, which is largely determined by the traffic flows at the test sites. We use matrix $F(\kappa )$ in (5) to denote the traffic flows on day $\kappa$.

5 $$\begin{aligned} F(\kappa )=\left[ \begin{array}{c} F_1(\kappa )\\ \vdots \\ F_i(\kappa )\\ \vdots \end{array} \right] =\left[ \begin{array}{cccc} f_{1,1}(\kappa )&{}f_{1,2}(\kappa )&{}\dots &{}f_{1,t^*}(\kappa )\\ \vdots &{}\vdots &{}\ddots &{}\vdots \\ f_{i,1}(\kappa )&{}f_{i,2}(\kappa )&{}\dots &{}f_{i,t^*}(\kappa )\\ \vdots &{}\vdots &{}\ddots &{}\vdots \end{array} \right] , \end{aligned}$$

where $F_i(\kappa )$ is the flow vector along road segment *i* on day $\kappa$; $f_{i,t}(\kappa )$ is the number of vehicles (per unit time) passing road segment *i* during interval *t* on day $\kappa$. Testing is scheduled on a daily basis, and in theory, the flow matrix is a function of date.

Traffic flow measurements can be obtained by various sensors, e.g., loop detectors^15^. However, there are a limited number of traffic flow sensors. Consequently, the method requires estimating the traffic flow on links with no traffic flow sensors. Origin-destination matrix estimation is often used to assess the demand for transport and impute flows on unequipped links^16^. In the absence of an accurate state-wide OD matrix, we use data from traffic flow sensors to estimate the flows on the nearby road segments. For the sake of simplicity, we use the imputation method in (6) to estimate the unknown flow values using the known.

The locations of traffic flow sensors are denoted as $X=\{x_1,x_2,\dots \}$, where $x_i=\!\!\hbox {[latitude, longitude]}$ is the location of road segment *i* that has a traffic flow sensor; the detected flow at $x_i$ on day $\kappa$ is denoted as $f(x_i,\kappa )$. Similarly, the set of locations of roads that do not have traffic flow sensors is denoted as $Y=\{y_1,y_2,\dots \}$; the actual flow of road segment *j* on day $\kappa$ is $f(y_j,\kappa )$. Note that $f(x_i,\kappa )$ and $f(y_j,\kappa )$ are traffic flow vectors, where $f(x_i,\kappa )=\left[ f_{i,1}(\kappa ),\dots ,f_{i,t^*}(\kappa )\right]$ and $f(y_j,\kappa )=\left[ f_{j,1}(\kappa ),\dots ,f_{j,t^*}(\kappa )\right]$. For any $y_j$, we find $U_j$ locations $x_{1},\dots ,x_{U_j}\in X$ that are closest to $y_j$. We estimate the flow at $y_j\in Y$ as below.

6 $$\begin{aligned}{} & {} {\hat{f}}(y_j,\kappa )=\overset{U_j}{\underset{i=1}{\sum }}0.5\left( 1-\frac{\Vert y_j-x_i\Vert }{\overset{U_j}{\underset{i=1}{\sum }}\Vert y_j-x_i\Vert }\right) f(x_i,\kappa ).\nonumber \\{} & {} \Vert x_i-y_j\Vert <\Vert x'-y_j\Vert , \forall 1\le i\le U_j,x'\in X \text{ and } x_i\ne x'. \end{aligned}$$

The estimated traffic flows are the weighted averages of flows of nearest segments with measurements and are regarded as elements of the flow matrix, as $f(y_i,\kappa )={\hat{f}}(y_i,\kappa )$.

We thus obtain $F(\kappa )$, which is a function of the day. Also, we can calculate the averaged traffic flow matrices ${\hat{F}}(Monday)$, $\dots$, ${\hat{F}}(Sunday)$ on the days (Monday-Sunday) of the week. Given a date to be scheduled, we find the traffic flow matrix that is on the same day of the week. Then, it will be used in the calculation of fitness values for GA-based optimisation.

**Figure 2:**
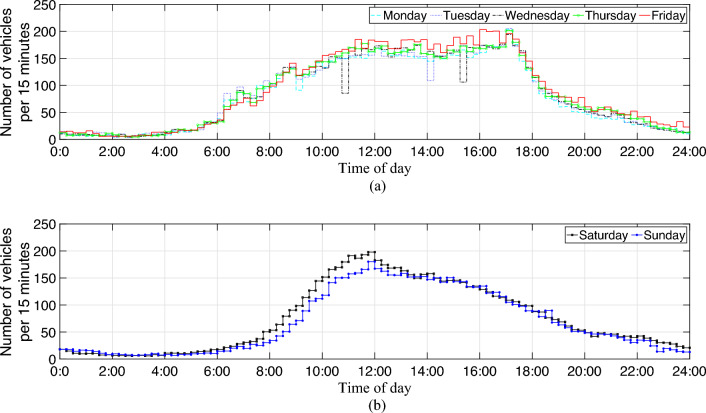
Temporal distribution of traffic flow per 15 min in 2019, Victoria, Australia. (**a**) Averaged traffic flows on weekdays (**b**) Averaged traffic flows on weekends.

The temporal distributions of the averaged weekday/weekend traffic flows in 2019, Victoria, Australia are shown in Fig. 2. The y-axis is the daily average number of vehicles per 15 min on roads with traffic flow sensors (see Fig. 7 displaying the roads that mainly include rural roads). It can be seen that the flows are high from 8am to 5pm during the weekdays.

### Characteristics of alcohol-related crashes

Drink drivers would not typically report drink driving themselves, and the police would also not know the origins, destinations, or paths of drink driver offenders. Data on traffic crashes are publicly available in some jurisdictions, and a proportion of them are related to alcohol. We use alcohol-related crash data to estimate drink driver path characteristics.

We have obtained traffic crash records in Victoria, Australia, which include the date, time, and location of the alcohol-related crashes. We divide the number of crashes in the day into $t^*$ intervals and present the drink driving patterns using matrix $C(\kappa )$ in (7).

7 $$\begin{aligned} C(\kappa )=\left[ \begin{array}{c} C_1(\kappa )\\ \vdots \\ C_i(\kappa )\\ \vdots \end{array} \right] =\left[ \begin{array}{cccc} c_{1,1}(\kappa )&{}c_{1,2}(\kappa )&{}\dots &{}c_{1,t^*}(\kappa )\\ \vdots &{}\vdots &{}\ddots &{}\vdots \\ c_{i,1}(\kappa )&{}c_{i,2}(\kappa )&{}\dots &{}c_{i,t^*}(\kappa )\\ \vdots &{}\vdots &{}\ddots &{}\vdots \end{array} \right] , \end{aligned}$$

where $c_{i,t}(\kappa )$ is the number of crashes during the *t*-th interval on road segment *i* on day $\kappa$. We endeavour that the spatial and temporal distributions of the synthetic crashes of simulated drink drivers in the simulation be similar to $C(\kappa )$ obtained from field crash data. See Section "Drink driving simulation" and Fig. 8 for further details.

**Figure 3:**
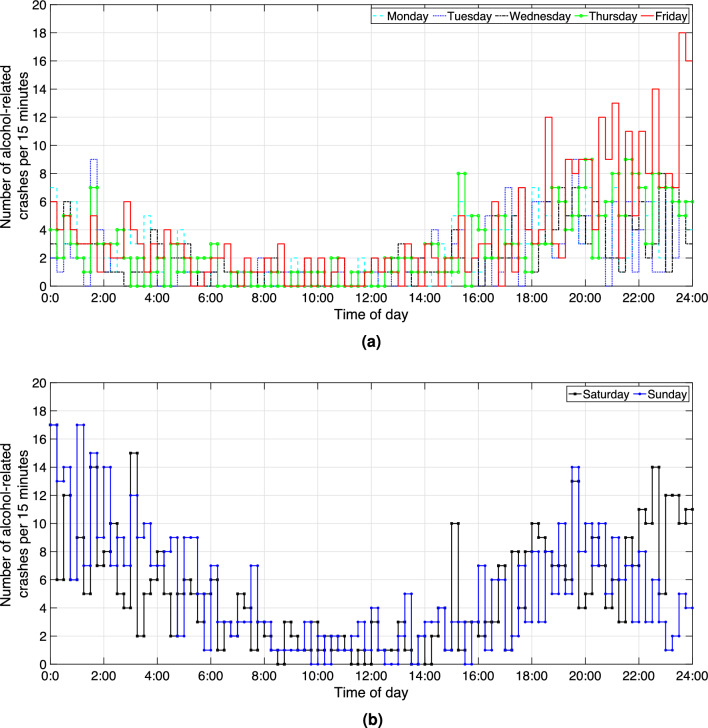
Number of alcohol-related crashes in Victoria, from July 2015 to June 2020. (**a**) Number of alcohol-related crashes on weekdays (**b**) Number of alcohol-related crashes on weekends.

The temporal distributions of alcohol-related crashes from July 2015 to June 2020 are presented in Fig. 3. The y-axis is the total number of alcohol-related crashes per 15 min and the x-axis is the time of the day. The crashes on weekdays are displayed in Fig. 3a and the crashes on weekends are plotted in Fig. 3b. It can be seen that the numbers of alcohol-related crashes are higher at night, after 5 pm (weekdays and weekends) and before 4 am (weekends). Additionally, there are more alcohol-related crashes on the weekends than on the weekdays.

### Traffic flow and random breath testing capacity at each site

Random breath tests are normally carried out by police. There are limited police resources at a single test site, and each test site has a limited testing capacity. The number of tests per minute at a test site has an upper bound of $\varsigma$. This means that the larger the traffic flow, the more likely drivers would not get tested.

We define that one test result is obtained if one driver is tested. If the number of passing vehicles per minute on a road segment with a test site is equal to or less than $\varsigma$, all drivers will be tested. If the number of vehicles per minute on the road is greater than $\varsigma$, then the likelihood of an arbitrary driver being tested decreases as the number of passing vehicles increases. Without loss of generality, we assume that $\varsigma =4/min$.

**Figure 4:**
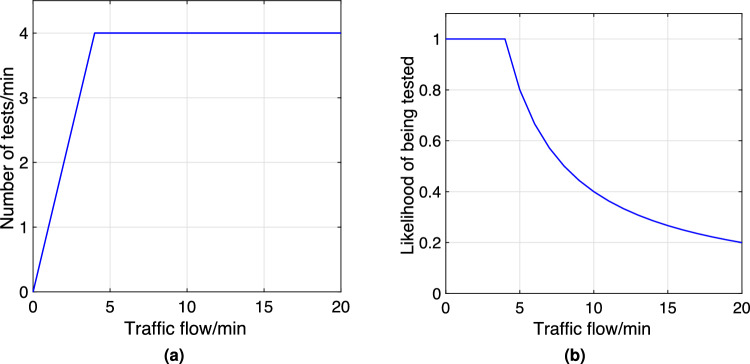
Number of RBT tests per min and likelihood of being tested under different traffic flows.

In Fig. 4a, in the low traffic flow regime, more tests are performed as there are more vehicles passing on the road. As the rate of passing vehicles reaches the capacity of the test site, the rate of tests remains at $\varsigma =4/min$. In Fig. 4b, all vehicles passing on the road are tested as long as the rate is below the capacity of the test site; the more vehicles there are on the road, the lower the likelihood of being tested. In practice, the likelihood is lower bounded as there is a finite number of vehicles on the road. We use (8) to calculate the likelihood $p_{i,t}(\kappa )$ of a driver being tested during the *t*-th interval on road segment *i* on day $\kappa$.

8 $$\begin{aligned} p_{i,t}(\kappa )= & {} \left\{ \begin{array}{cc} 1 &{}\text{ if } \quad f_{i,t}(\kappa )\frac{t^*}{1440}\le \varsigma ;\\ \frac{1440\varsigma }{t^*\cdot f_{i,t}(\kappa )} &{}\text{ if } \quad f_{i,t}(\kappa )\frac{t^*}{1440}>\varsigma . \end{array} \right. \end{aligned}$$

Note that $f_{i,t}(\kappa )$ is the number of vehicles along road segment *i* during interval *t* on day $\kappa$; $f_{i,t}(\kappa )\frac{t^*}{1440}$ is the number of vehicles during a one-minute interval. The number of vehicles per minute is assumed to be constant during each interval.

In comparison with the number of passing vehicles, the number of drink drivers is small. In GA-based optimisation, the likelihood in (8) is used to calculate the fitness values. In the simulation, the likelihood determines whether drink drivers are tested. The performance of testing schedules can be verified from simulations. In this way, evaluations of scheduled performances will be reliable and can be used in optimisation.

### Fitness function

The goal of roadside alcohol testing is to increase general deterrence. We measure the testing-delivered deterrence by the number of tests and the number of observing drivers. Note that the observing drivers are general drivers (sober or drunk) who drive past a test site but do not get tested. The overall deterrence can be measured by a weighted sum of the numbers of positive tests, negative tests, and observing drivers.

Positive test results can be approximated from drink drivers being tested at a test site. We can find the expected number ${\bar{\Lambda }}(\kappa )$ of positive results by (9).

9 $$\begin{aligned} {\bar{\Lambda }}(\kappa )=\underset{i}{\sum }\underset{t=1}{\overset{t^*}{\sum }}s_{i,t}(\kappa )\left( \underset{u}{\sum }q^u_{i,t}(\kappa )p_{i,t}(\kappa )\right) , \end{aligned}$$

where $q^u_{i,t}(\kappa )=1$ or 0 denotes whether the *u*-th drink driver travels along road segment *i* at time interval *t* on day $\kappa$ or not. Note that (9) might lead to insignificant overestimation because in reality a drink driver will be stopped in the first encountered RBT test site. Whereas (9) counts the number of positive test results based on the number of test sites on the routes of drink drivers.

The routes of drink drivers are difficult to estimate. Thus, we strike a simple proxy by relating the routes of drink drivers to the historically aggregated number of alcohol-related crashes. So, we replace $\underset{u}{\sum }q^u_{i,t}(\kappa )$ with $c_{i,t}(\kappa )$ in (9) and obtain the approximation of the expected number of positive tests on day $\kappa$, ${\bar{P}}(\kappa )$, as:

10 $$\begin{aligned} {\bar{P}}(\kappa )=E(P(\kappa ))=\underset{i}{\sum }\underset{t=1}{\overset{t^*}{\sum }}s_{i,t}(\kappa )c_{i,t}(\kappa )p_{i,t}(\kappa ), \end{aligned}$$

where $E(\cdot )$ is the expected value.

Traffic flows traversing through test sites determine the number of total tests. In this paper, we consider that drivers who do not drink can be tested multiple times. We estimate the expected number of total tests ${\bar{Q}}(\kappa )$ by (11) below.

11 $$\begin{aligned} {\bar{Q}}(\kappa )=E(Q(\kappa ))=\underset{i}{\sum }\underset{t=1}{\overset{t^*}{\sum }}s_{i,t}(\kappa )f_{i,t}(\kappa )p_{i,t}(\kappa ). \end{aligned}$$

After the number of total tests is obtained, we can find the difference between traffic flows traversing through the test sites and the number of tests. This quantity would be the number of observing drivers. Below, we calculate the number ${\bar{R}}(\kappa )$ of observing drivers.

12 $$\begin{aligned} {\bar{R}}(\kappa )=E(R(\kappa ))=\underset{i}{\sum }\underset{t=1}{\overset{t^*}{\sum }}s_{i,t}(\kappa )f_{i,t}(\kappa )-{\bar{Q}}(\kappa ). \end{aligned}$$

General deterrence is measurable by the number of tests (positive and negative) plus observing drivers. Based on $P(\kappa )$, $Q(\kappa )$, and $R(\kappa )$, we introduce (13) to define fitness value $V(\kappa )$.

13 $$\begin{aligned} V(\kappa )=\alpha P(\kappa )+\beta Q(\kappa )+\gamma R(\kappa ), \end{aligned}$$

where $\alpha >0$, $\beta >0$, and $\gamma >0$ are constant weights. We can obtain ${\bar{V}}(\kappa )$ as below.

14 $$\begin{aligned} {\bar{V}}(\kappa )= & {} E(V(\kappa ))=E(\alpha P(\kappa )+\beta Q(\kappa )+\gamma R(\kappa ))\nonumber \\= & {} \alpha {\bar{P}}(\kappa )+\beta {\bar{Q}}(\kappa )+\gamma {\bar{R}}(\kappa )\nonumber \\= & {} \alpha \underset{i}{\sum }\underset{t=1}{\overset{t^*}{\sum }}s_{i,t}(\kappa )c_{i,t}(\kappa )p_{i,t}(\kappa )+(\beta -\gamma )\underset{i}{\sum }\underset{t=1}{\overset{t^*}{\sum }}s_{i,t}(\kappa )f_{i,t}(\kappa )p_{i,t}(\kappa )+\gamma \underset{i}{\sum }\underset{t=1}{\overset{t^*}{\sum }}s_{i,t}(\kappa )f_{i,t}(\kappa ),\nonumber \\= & {} \underset{i}{\sum }\underset{t=1}{\overset{t^*}{\sum }}s_{i,t}(\kappa )\Bigl (\alpha p_{i,t}(\kappa )c_{i,t}(\kappa )+\bigl (\beta p_{i,t}(\kappa )+\gamma \left( 1-p_{i,t}(\kappa )\right) \bigl )f_{i,t}(\kappa )\Bigl ). \end{aligned}$$

We use (14) to obtain the fitness values of RBT schedules. It is a weighted sum of historically aggregated alcohol-related crashes and traffic flows. Note that there is no direct way to obtain the values for $\alpha$, $\beta$, and $\gamma$. In Section "RBT performance results", the values are obtained by trial and error. The optimisation approach in this study is used as a guide for policymakers on RBT testing. In practice, by reasonably increasing the value of $\alpha$ or decreasing the value of $\beta$, we can use the method to increase the number of positive results out of the same number of total tests or budget. Alternatively, by reasonably decreasing the value of $\alpha$ or increasing the value of $\beta$, we can increase the number of total tests while capturing relatively the same number of positive results.

## Optimisation method

In this section, we present a GA-based optimisation method and use *k*-medoids clustering to partition days based on the temporal occurrence of alcohol-related crashes. Given testing schedules, we use the fitness function in (14) to evaluate the testing schedules, and use mutation and crossover to optimise the testing schedules.

### GA-based optimisation

**Figure 5:**
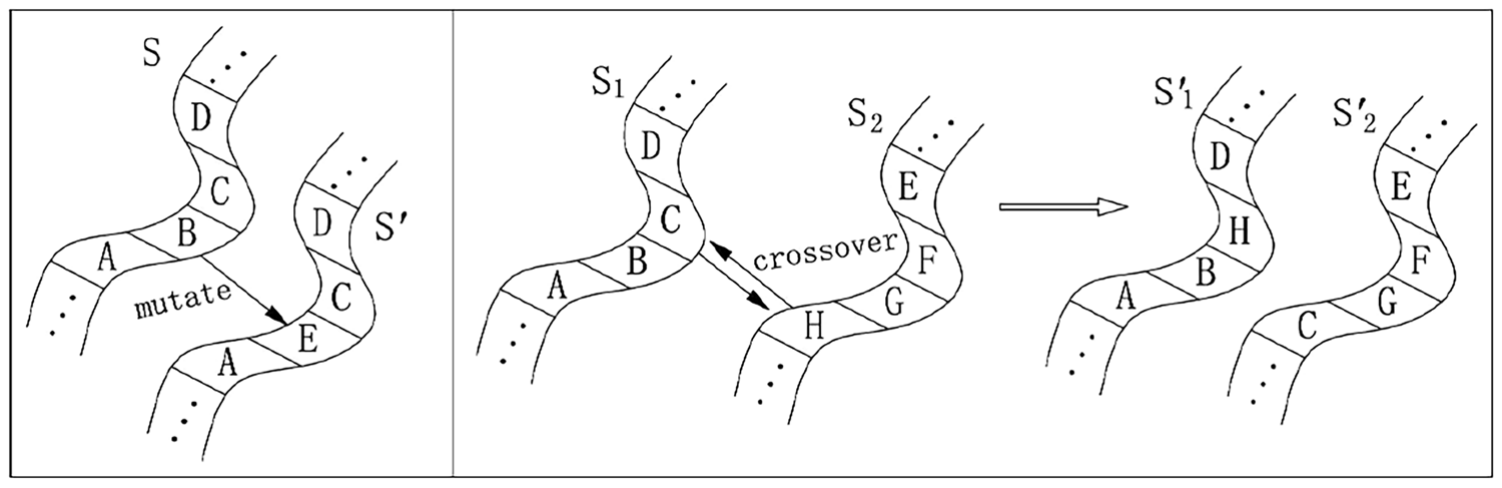
Mutation and crossover of genes.

The testing schedules can be represented by a sequence of testing states (see Eq. (2)). The variables are discrete and GA-based optimisation is credible as a solution (e.g.,^17,18^). Mutation and crossover can be used to generate new genes (test schedules in the proposed method). For example in Fig. 5, the genes *S*, $S_1$, and $S_2$ are denoted by *ABCD*, *ABCD*, and *HGFE*, respectively (*A*-*H* each denotes a test site). By mutation, *B* turns into *E* and *S* turns into a new gene $S'$, as is shown on the left of Fig. 5. By crossover, $S_1$ and $S_2$ exchange *C* and *H*, turning into $S'_1$ and $S'_2$, as is shown on the right of Fig. 5. Based on the current schedule, new schedules are obtained by mutation and crossover. Different RBT schedules have different fitness values, and mutation and crossover do not necessarily produce new schedules with higher fitness values. As schedules with higher fitness values are obtained and the fitness values continue to increase, the optimal schedule will be found with the highest fitness value. We regard a test schedule as a gene, and GA is used to obtain the optimal test schedule.

A testing schedule can have multiple test sites, $g_w(\kappa )$,$1\le w\le N$ on day $\kappa$; such a notion contains a road number, start- and finish-time for testing. By (3), we have:

$$\begin{aligned} {\tilde{S}}(\kappa )=\underset{w=1}{\overset{N}{\uplus }}g_w(\kappa ), \end{aligned}$$

where $g_w(\kappa )=\{{\bar{t}}_w,{\underline{t}}_w,i_w\}$ is a test site that starts from ${\bar{t}}_w$ and lasts till ${\underline{t}}_w$ along road $i_w$ on day $\kappa$. Given $\Phi _1$ RBT schedules, we can obtain new sites and schedules by mutation and crossover, which randomise not only the time and duration of RBT sites but also their locations. See below the pseudo-code for GA RBT optimisation. Note that we set  $N_{\max }=58,\;t_{\min }=1,\;t_{\max }=3,\;\Delta =58,\;\Phi _1=100,\;\Phi _2=30,\;\Phi _3=34,\;\Phi _4=50,\;p_{\textrm{E}}=40\%,\;p_{\textrm{S}}=30\%,\;p_{\textrm{G}}=30\%,\;p_{\textrm{e}}=50\%$ , and $\;p_{\textrm{s}}=50\%$ in Algorithm 1. The GA-related parameters are tuned based on trial and error procedure.

The parameters $p_{\textrm{E}}$, $p_{\textrm{S}}$, $p_{\textrm{G}}$, $p_{\textrm{e}}$, and $p_{\textrm{s}}$ control the possibility of an operation in Algorithm 1, such that after a schedule is selected for mutation, one of the three operations of ‘Extend’, ‘Split’, and ‘Generate’ will be executed. The possibilities of these operations are $p_{\textrm{E}}$, $p_{\textrm{S}}$, and $p_{\textrm{G}}$ with $p_{\textrm{E}}+p_{\textrm{S}}+p_{\textrm{G}}=1$. When the operation ‘Extend’ takes place, a random test site from the selected schedule will be picked. The possibility that the test site gets extended by starting one interval earlier is $p_{\textrm{e}}$. Similarly, the possibility that the test site gets extended by finishing one interval later is $1-p_{\textrm{e}}$. With the operation ‘Split’, a random test site will be picked on the premise that its duration is at least twice the minimal testing duration ($t_{\min }$) and divided into two parts. Another location will be randomly picked where a new test site can be set up. The possibility that the new test site duration is the same as the first split part is $p_{\textrm{s}}$, and accordingly, the possibility that the new test site duration is the same as the second split part is $1-p_{\textrm{s}}$.

### Clustering of days based on temporal occurrence of alcohol-related crashes

Drink driving characteristics such as departure time and trips origins and destinations differ from sober driving. We consider that drink driving has relevance to temporal and spatial distributions of alcohol-related crashes. We obtained data on alcohol-related crashes, which includes latitudes and longitudes and the time and date of the crashes. The alcohol-related crash matrices will be used to account for drink driving to evaluate the performance of the testing schedules.

On day $\kappa$, the crash vectors are denoted as $\psi _{\kappa }=\left[ \psi _{\kappa ,1},\dots ,\psi _{\kappa ,24}\right]$, where $\psi _{\kappa ,h}$ is the number of alcohol-crashes from $(h-1):00$ to *h* : 00 on day $\kappa$. There are 24 attributes for the crash vector of any day. We use *k*-medoids clustering to partition the set $\Psi =\{\psi _1,\psi _2,\dots \}$ into *k* clusters $A,B,\dots$. Under the clustering function $z(\cdot )$, a data point $\psi _\kappa \in \Psi$ is assigned to set $\Psi ^*_k$, whose medoid is $\psi ^*_k=z(\psi _\kappa )$. We consider $N_\text {c}=14$ types of days, which are listed in Table 1 as Holiday Mondays - Sundays and the Non-Holiday days. After *K* clusters are obtained, we use $o(\cdot )$ as a function from date type to cluster such that we are able to assign date type *j*, $1\le j\le N_\text {c}$, to cluster *o*(*j*). $m_{kj}$ is the number of data points of date type *j* that is assigned to cluster $\Psi _k$. We use Algorithm 2 and results show the best performance with $K=4$ clusters.

**Algorithm 1:**
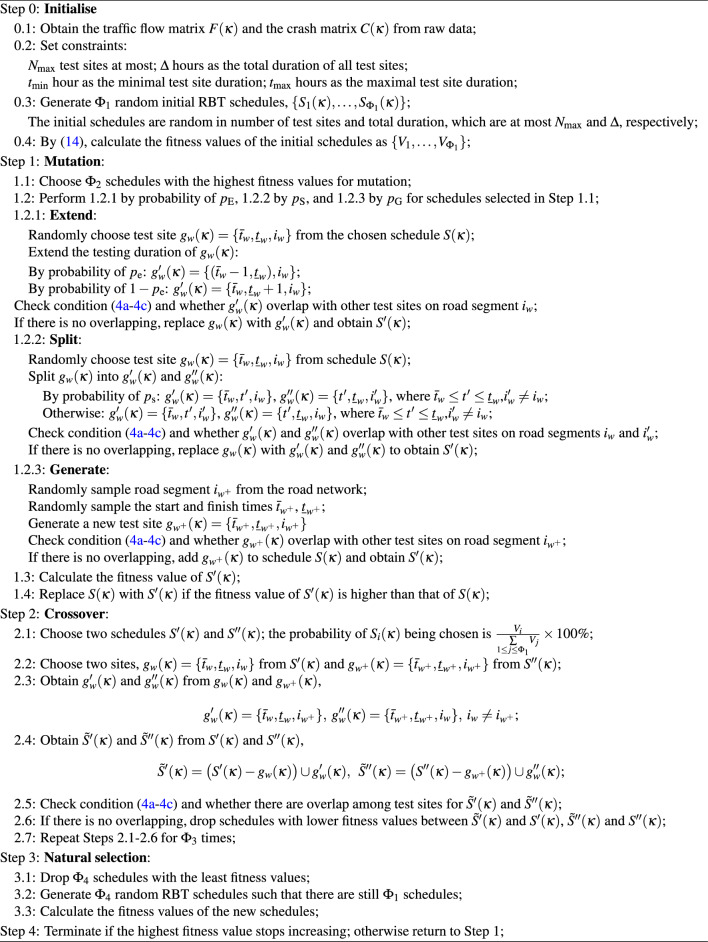
GA for RBT Optimisation

**Algorithm 2:**
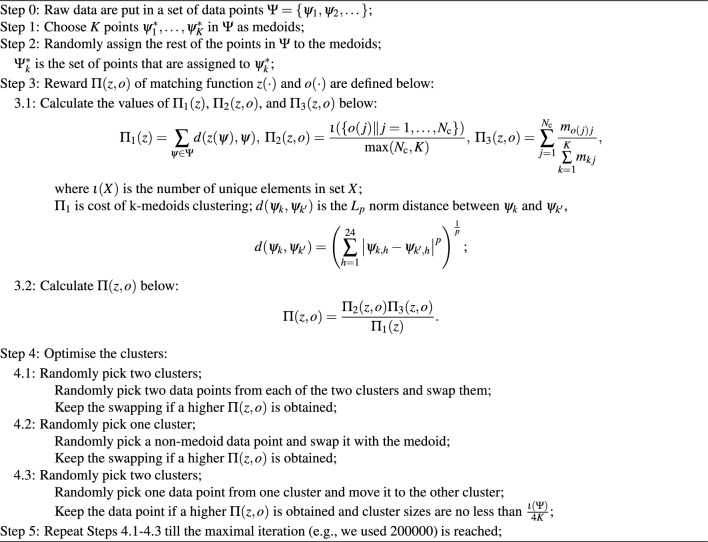
*k*-medoids Clustering

**Table 1:** The association among the $K=4$ clusters and $N_\text {c}=14$ day types.

Dates	Holiday						
Clusters	Monday	Tuesday	Wednesday	Thursday	Friday	Saturday	Sunday
A	$\overset{83.4\%}{{\checkmark }}$	10.5%	0	0	12.5%	0	0
B	8.3%	$\overset{79.0\%}{{\checkmark }}$	0	16.7%	$\overset{87.5\%}{{\checkmark }}$	$\overset{85.7\%}{{\checkmark }}$	0
C	8.3%	0	20.0%	8.3%	0	0	$\overset{100\%}{{\checkmark }}$
D	0	10.5%	$\overset{80.0\%}{{\checkmark }}$	$\overset{75.0\%}{{\checkmark }}$	0	14.3%	0

	Non-Holiday						
	Monday	Tuesday	Wednesday	Thursday	Friday	Saturday	Sunday
A	10.9%	3.7%	10.1%	9.1%	$\overset{75.1\%}{{\checkmark }}$	$\overset{62.2\%}{{\checkmark }}$	11.3%
B	$\overset{72.6\%}{{\checkmark }}$	3.7%	10.7%	11.2%	9.1%	13.3%	12.2%
C	6.3%	7.4%	16.8%	$\overset{70.6\%}{{\checkmark }}$	6.7%	14.8%	$\overset{66.9\%}{{\checkmark }}$
D	10.2%	$\overset{85.2\%}{{\checkmark }}$	$\overset{62.4\%}{{\checkmark }}$	9.1%	9.1%	9.7%	9.6%

Note that we set $K=4$ and obtain four clusters $\Psi ^*_1,\dots ,\Psi ^*_4$ (denoted as *A*, *B*, *C*, and *D*) by Algorithm 2. Algorithm 2 guarantees the cluster sizes to be no smaller than $\frac{\iota (\Psi )}{4K}$ to make sure that the clusters contain adequate data points and are statistically significant. From the four clusters, we obtain the cluster crash matrices $C^A$, $\dots$, $C^D$ for clusters *A*, $\dots$, *D*, respectively.

We consider types of dates including holiday and non-holiday Monday-Sunday and obtain Table 1. Each type of date most likely belongs to one cluster (marked by ${\check{mark}}$). Thus, given any date to be scheduled, we are able to identify one type of date from the 14 date types in Table 1 and a matching cluster. We then obtain the cluster crash matrix from data points in the cluster. By combing the matrix with the daily flow matrix $F(\kappa )$, we determine RBT-opt1 and RBT-det schedules detailed in Sections "GA-based optimisation" and "Benchmark methods".

**Figure 6:**
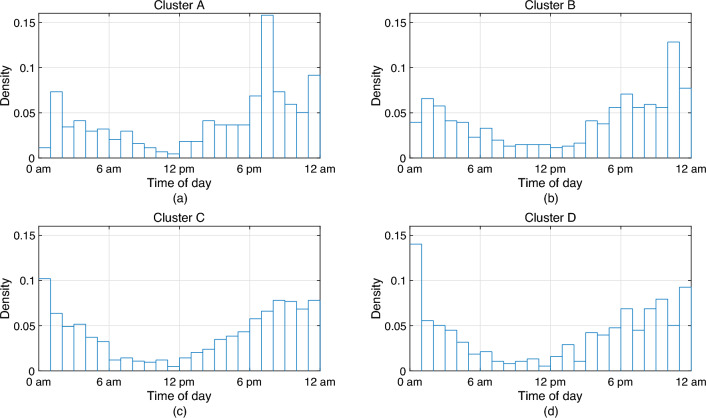
Temporal distributions of alcohol-relted crashes in the 4 clusters.

We use the alcohol-related crash data in Victoria, Australia for clustering of days based on the temporal occurrence of drink driving crashes from July 1st, 2015 to June 30th, 2020. The data are represented by the number of crashes in 24 h. There are 2256 cases of alcohol-related crashes in 1189 days when there is at least one alcohol-related crash. The number of clusters needs to be reasonably small such that there is an adequate number of data points in each cluster and the cluster crash matrices are statistically significant. We divide the data points into 4 clusters such that there are at least 75 data points in each cluster. The clusters’ temporal distributions of the alcohol-related crashes are shown in Fig. 6a–d.

Drink driving has different characteristics on different types of dates, such as weekdays, weekends, and public holidays. The clusters *A*, $\dots$, *D* include particular date types. Given a date and whether it is a weekday, weekend, or public holiday, we can decide whether such a date belongs to a certain cluster using Table 1. We then use the crash matrices obtained from the cluster to describe drink driving on the day. Ultimately, we can proceed to the scheduling optimisation.

## Data and numerical experiments

We investigate the performance of the proposed RBT scheduling method using the road network of Victoria, Australia. Crash data and traffic flow data are crucial in determining RBT schedules and analysing performances.

*Road Network:* The road network of VIC, Australia in this paper is extracted from the VIC OpenStreetMap file. The network contains 115596 links.

*Crash Data:* The crash data were obtained from VicRoads Open Data. Each alcohol-related crash was recorded with a time, date, and location (latitude and longitude). A total number of 2256 alcohol-related crashes were found from July 2015 to June 2020, and they are projected to 2215 links in the road network.

*Traffic Flow Data:* Traffic flow data in 2019 is obtained from Data Vic. Each traffic flow was recorded with a time interval (15 min), day of the week, and a geometric curve (points of latitudes and longitudes). A total number of 6317 raw flows are found. After imputation, they are projected onto 115596 links in the road network.

**Figure 7:**
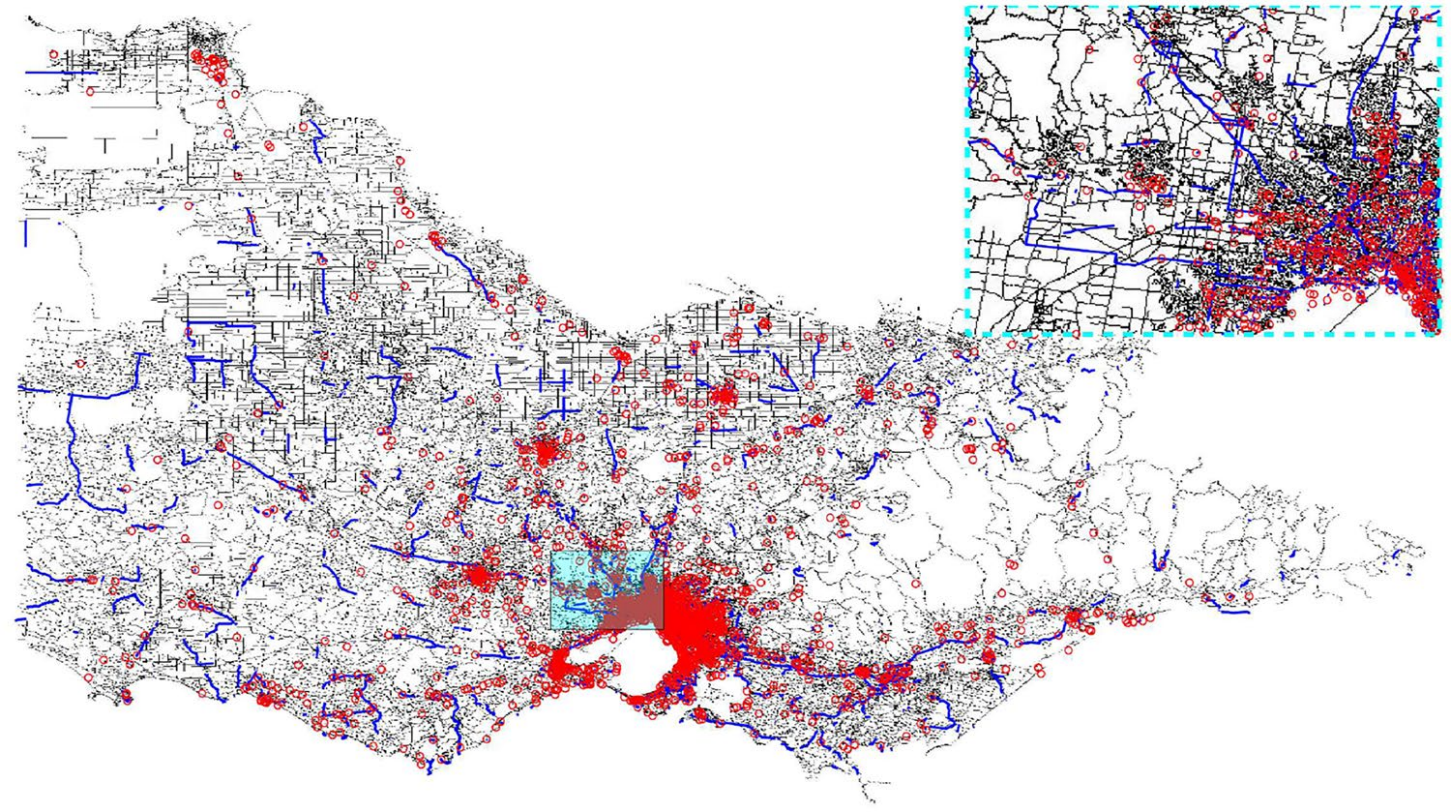
Network of Victoria, Australia. Red circles denote the spatial distribution of alcohol crashes, and blue lines show links with traffic sensors. Part of Melbourne is expanded in the upper right corner (Map data: https://www.openstreetmap.org).

The road network is shown in Fig. 7. The latitudes and longitudes of traffic flows and alcohol-related crashes are projected on the network, which is denoted by bold blue lines and red circles in Fig. 7. It can be seen that a large proportion of road segments in Victoria do not have records of traffic flows or alcohol crashes, even for urban areas in the upper right corner representing part of metropolitan Melbourne.

### Benchmark methods

In Section "GA-based optimisation", we have proposed a GA-based optimisation approach to obtain the optimised RBT schedule by the fitness function presented in Section "Fitness function". The method uses the crash matrix and traffic flow matrix to optimise the RBT schedules. We use four types of RBT schedules for benchmarking.

- RBT-opt1: First, we obtain the cluster crash matrix ($C^A-C^D$) from the cluster that matches the date to be scheduled. Using the flow matrix and the matching cluster crash matrix, we use the GA-based method to get the optimised RBT schedules.
- RBT-opt2: We obtain the overall crash matrix ${\hat{C}}$ from the crash data. Then, we use the GA-based method on the flow matrix and the overall crash matrix to get the optimised RBT schedules (RBT-opt2) for the considered period. This benchmark method is considered to evaluate the effect of clustering on the overall method.
- RBT-rand: Randomly sample locations from the road network and start/finish times to obtain the random RBT schedules while satisfying (4a-4c). The times and the test site locations have the same probabilities of being chosen.
- RBT-det: We follow the following steps to obtain the RBT-det schedule.
  - Modify the crash matrix: Given day $\kappa$, we first find the matching traffic flow matrix ${\hat{F}}(\kappa )=[{\hat{f}}_{i,t}(\kappa )]$ with ${\hat{f}}^*(\kappa )=\underset{i,t}{\max }{\hat{f}}_{i,t}(\kappa )$. Then, based on ${\hat{F}}(\kappa )$ and the overall crash matrix ${\hat{C}}$, we obtain the modified crash matrix ${\bar{C}}(\kappa )=[{\bar{c}}_{i,t}(\kappa )]$, where ${\bar{c}}_{i,t}(\kappa )={\hat{c}}_{i,t}(\kappa )+\frac{2{\hat{f}}_{i,t}(\kappa )}{{\hat{f}}^*(\kappa )}$.
  - Constraints on the RBT-det schedule: For simplification, we set the duration of a test site as 1 h and 15 min.
  - Obtain the RBT-det schedule: We calculate and find the benchmark schedule $S^{\text {det}}(\kappa )=\{g_1(\kappa ),\dots ,g_w(\kappa ),\dots \}$ by (15), where the *w*-th test site is denoted as $g_w(\kappa )=\{{\bar{t}}_w,{\underline{t}}_w,i_w\}$.
    15 $$\begin{aligned} S^{\text {det}}(\kappa )= & {} \underset{{{(4a-4c),}}}{\underset{{{\bar{t}}_w,{\underline{t}}_w,i_w}}{\max }}\;\underset{w\ge 1}{\sum }\;\overset{{\underline{t}}_w}{\underset{t\ge {\bar{t}}_w}{\sum }}{\bar{c}}_{i_w,t}(\kappa ). \end{aligned}$$

### Drink driving simulation

We use the road network in Fig. 7 to simulate drink driving. We assume that drink drivers drive from random origins to random destinations along the shortest travel time paths and the departure times are generated from the corresponding cluster (see Fig. 6). The traffic flow matrix in Section "Traffic flow imputation" is used to determine the likelihood of drivers being tested. As soon as the RBT schedules are implemented, drink drivers will be tested and positive results will be collected. We use start times and the assumed paths to determine the locations of drink drivers at any given time. Assume that a drink driver starts driving at $\tau _1$ from node $i_1$ to node $i_2$ (link $i_1\rightarrow i_2$) with speed $v_{i_1i_2}$. The path is $i_1\rightarrow i_2\rightarrow \dots \rightarrow i_u$. Intuitively, the time interval when the drink driver is on link $i_1\rightarrow i_2$ is $(\tau _1,\tau _1+\frac{l_{i_1i_2}}{v_{i_1i_2}})$. In a general form, the time interval when the drink driver is on link $i_\nu \rightarrow i_{\nu +1}$ is $(\tau _1+\underset{u=1}{\overset{\nu -1}{\sum }}\frac{l_{i_ui_{u+1}}}{v_{i_ui_{u+1}}},\tau _1+\underset{u=1}{\overset{\nu }{\sum }}\frac{l_{i_ui_{u+1}}}{v_{i_ui_{u+1}}})$.

**Figure 8:**
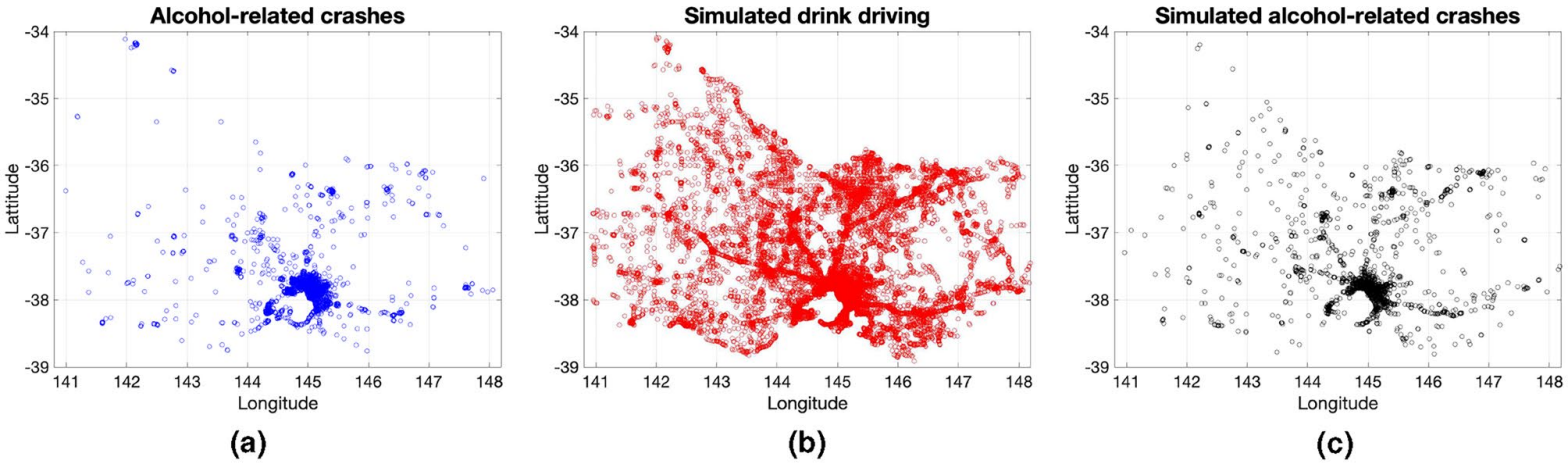
Comparison among spatial distributions. A point in (**a**), (**b**), and (**c**) respectively denotes one alcohol-related crash, simulated drink driver, and one simulated alcohol-related crash over the period from July 1st, 2015 to June 30th, 2020. (**a**) Ground truth alcohol-related crashes; (**b**) Simulated drink driving traces; and (**c**) Simulated alcohol-related crashes.

Figure 8 illustrates the spatial distributions of ground truth alcohol-related crashes (2256 crashes over the 5-year period), simulated traces of drink driving, and simulated alcohol-related crashes. In the simulation, the average length per trip is 8.9 km. It can be seen that the simulated crashes in Fig. 8c have a similar spatial distribution to that of the ground truth alcohol-related crashes in Fig. 8a. Note that traces of drivers are shown as dots in Fig. 8b.

According to a survey released by^19^, the total yearly driving distance is about $6.36\times 10^{10}$ kilometers, which is $1.74\times 10^8$ km per day. Given that the average distance is 8.9km per trip, according to^20^, there are roughly $2\times 10^7$ car trips per day. In addition, there were 2256 alcohol-related crashes over the five-year period, and there are about 1.24 alcohol-related crashes on an average day.

### RBT performance results

In this section, we use simulation experiments to demonstrate the performances of testing schedules. There are 2256 alcohol-related crashes in 1189 days from July 2015 to June 2020. Each day is a data point, and the points are classified into 4 clusters, as shown in Fig. 6.

Given the introduced procedure, we are able to simulate a large number of drink drivers over a period of time in a road network. We assume the vehicle speed is 50km/h, and we have $\tau \sim \{\epsilon _A-\epsilon _D\}$ for the start time $\tau$ of drink drivers. We use the temporal distribution from the cluster ($A-D$) that matches the given date to generate drink driving. To accurately capture the traffic flow data variations in the simulation, we use ${\hat{F}}(Monday)$, $\dots$, ${\hat{F}}(Sunday)$ in Section "Traffic flow imputation" to represent traffic flows on different days. We consider RBT schedules for the time period from September 23rd, 2022 to September 29th, 2022, which includes a public holiday (23rd, Friday), two weekends (24th and 25th, Saturday and Sunday), and four weekdays (from 26th to 29th, Monday to Thursday). For each day during the considered time period, we find a cluster that matches the date type, as shown in Table 1. Therefore, we have that $\text{23rd, }\;\text{24th, }\;\text{26th }\in B$, $\text{25th, }\;\text{29th }\in C$, and $\text{27th, }\;\text{28th }\in D$. We obtain the cluster crash matrix from the matching cluster, which is joined by the traffic flow matrix to calculate fitness values and determine the likelihood of drink driving being tested at the test sites. In the computation of RBT-opt1 and RBT-opt2 schedules, the optimisation continues until the fitness values stop changing. The coefficients for the fitness function are chosen as $\alpha =0.999994;\; \beta =10^{-5};\;\gamma =-4\times 10^{-6}$. The constraints are $N_{\max }=58$, $t_{\min }=1$ [hr], $t_{\max }=3$ [hr], and $\Delta =58$. The performances of optimised and benchmark RBT schedules are listed in Table 2.

**Table 2:** Performance of the RBT schedules.

	Number of positive results				Percentage of trip traveled (%)			
	RBT-det	RBT-opt1	RBT-opt2	RBT-rand	RBT-det	RBT-opt1	RBT-opt2	RBT-rand
23rd, Friday	39	70	10	15	57.4	51.1	75.7	40.8
24th, Saturday	13	29	28	15	61.8	51.7	45.9	26.8
25th, Sunday	7	61	40	17	54.6	43.3	42.5	49.5
26th, Monday	12	78	127	11	73.6	49.8	47.2	47
27th, Tuesday	16	31	46	6	59.6	58.7	52.4	59.6
28th, Wednesday	27	88	61	17	41.9	40.6	56.1	46
29th, Thursday	41	31	59	9	51.8	57.2	55.4	60.1
Average	22	55	53	13	57	50	54	47
% improvement	Baseline	150	140.9	− 40.9	Baseline	12.3	5.3	17.5

	Number of tests				Number of observing drivers			
	RBT-det	RBT-opt1	RBT-opt2	RBT-rand	RBT-det	RBT-opt1	RBT-opt2	RBT-rand
23rd, Friday	6626	10543	5912	7507	4347	1659	980	8250
24th, Saturday	4252	8890	5420	8442	680	3381	1821	10597
25th, Sunday	5970	9021	8210	8019	2897	3454	5147	7964
26th, Monday	6489	9895	6826	7665	3396	3127	3185	8332
27th, Tuesday	6181	10233	6798	9719	6391	6255	1867	13963
28th, Wednesday	6160	10152	6928	7738	6703	4154	2705	9529
29th, Thursday	6636	8609	5192	9722	5779	2129	1167	13499
Average	6045	9620	6469	8402	4313	3451	2410	10305
% improvement	Baseline	59.1	7	39	Baseline	− 20	− 44.1	138.9

The units for the number of positive results, tests, and observing drivers are drivers. Note that RBT-opt1 and RBT-opt2 denote the optimised schedules using the cluster crash matrices and the overall crash matrix. RBT-det denotes the deterministic schedules, and RBT-rand denotes the random schedules. The improvement is calculated using RBT-det as the baseline. The negative values of improvement (in %) denote worse performance compared to the baseline method.

The RBT-det schedules are used as the baselines, and positive results, total tests, and observing drivers are used to evaluate the performances of RBT-opt1 and RBT-opt2. Improvement is presented in percentages and higher values represent more desirable performances. Note that we have added the percentage of trip traveled for offenders in the table. The definition is given below.

$$\begin{aligned} \text{ Percentage } \text{ of } \text{ trip } \text{ traveled }=\frac{1}{M}\underset{m}{\sum }\frac{\text{ distance } \text{ traveled } \text{ by } \text{ offender } \textit{ m } \text{ before } \text{ being } \text{ captured } \text{ at } \text{ an } \text{ RBT } \text{ site }}{\text{ distance } \text{ from } \text{ origin } \text{ to } \text{ destination } \text{ of } \text{ offender } \textit{ m }}\times 100\%, \end{aligned}$$

where there are *M* offenders in total. The sooner an offender is caught, the less likely there will be an alcohol-related road accident. Therefore, the lower the percentage is, the more effective the schedule is.

As observed in Table 2, on average, the random and deterministic RBTs capture 13 and 22 positive results and 8402 and 6045 tests in total. In comparison, the optimised schedules (RBT-opt1 and RBT-opt2) are able to capture 55 and 53 positive results and 9620 and 6469 overall tests during the considered period. The RBT-opt1 and RBT-opt2 methods result in lower numbers of observing drivers compared to the RBT-det baseline schedules; whereas RBT-rand schedules have averaged 10305 observing drivers, which is much higher. The percentages of trips traveled are also shown in Table 2, and the numbers are around 50$\%$ of the trip length and they are highest with the RBT-det schedules. This value measures what percentage of their trip an offender has traveled before being tested by a test site. Intuitively, the aim is to detect an offender sooner en route as any offender is a moving safety hazard. Note that the fitness function does not take the percentage travelled into account. Nevertheless, the optimised schedules have lower percentages on average while capturing more positive results.

**Figure 9:**
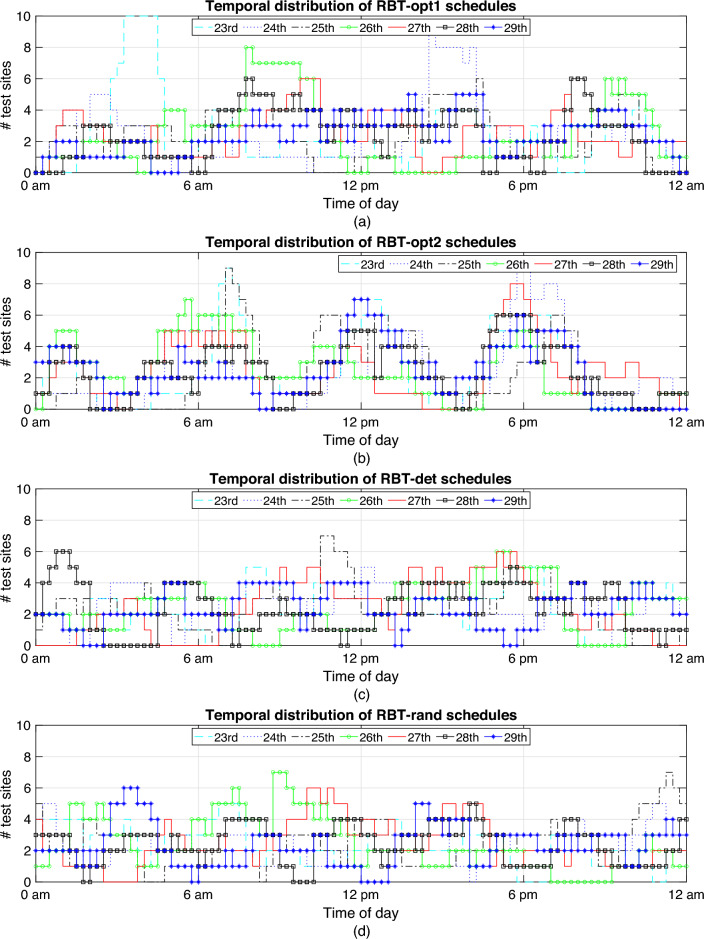
Temporal Distribution of the RBT schedules: (**a**) RBT-opt1; (**b**) RBT-opt2; (**c**) RBT-det; and (**d**) RBT-rand.

**Figure 10:**
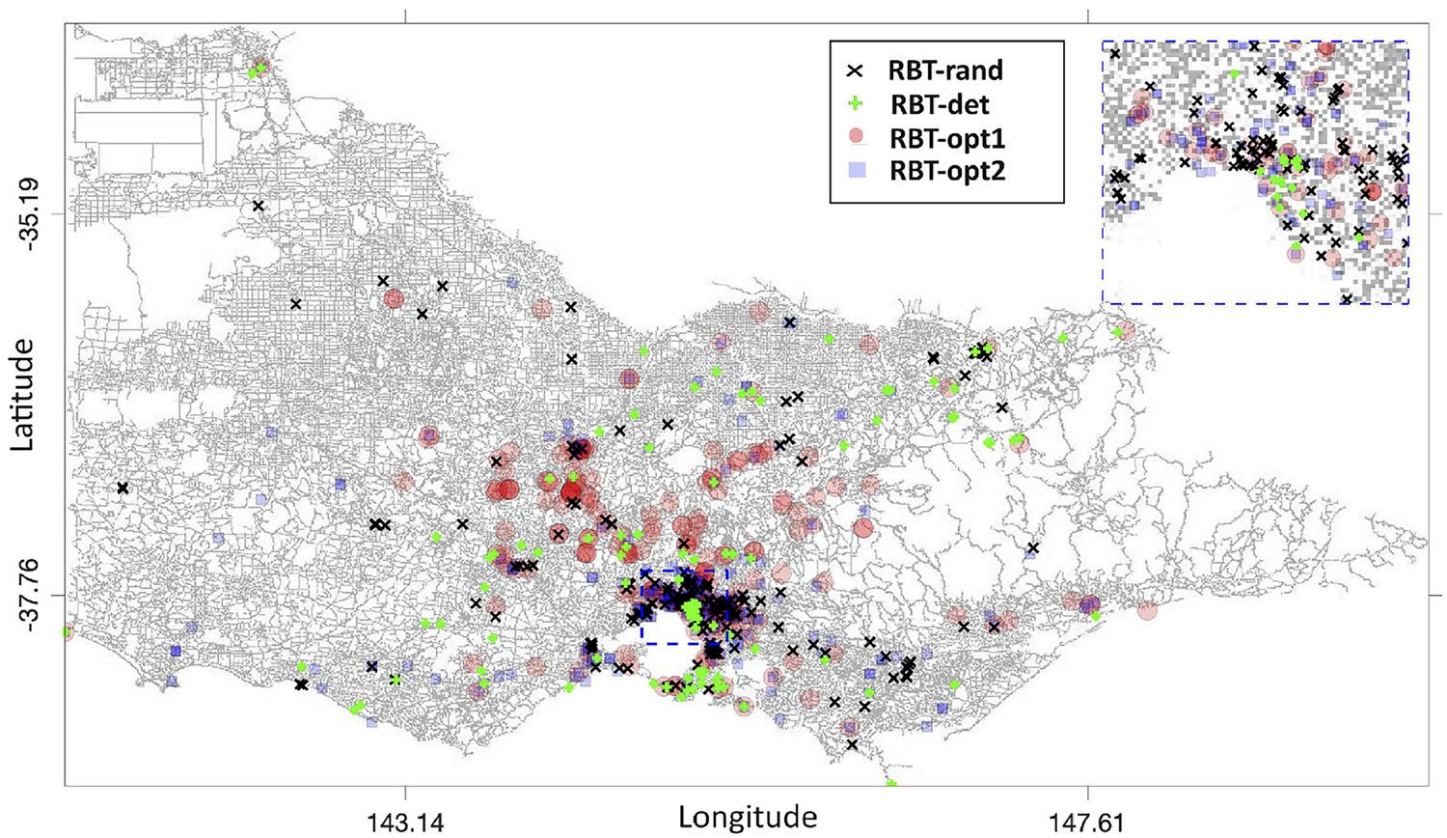
Black $\times$ denotes locations of RBT-rand schedules; green $+$ denotes locations of RBT-det schedules; red $\bigcirc$ denotes RBT-opt1 schedule; and blue $\Box$ denotes RBT-opt2 schedule (Map data: https://www.openstreetmap.org).

The temporal and spatial distributions of the four benchmark RBT sites during the considered period are shown in Figs. 9 and 10. The start/finish times of RBT-opt1 schedules in Fig. 9a are less concentrated than those in Fig. 9b, as they are centred around 9am and 9pm. The times of RBT-det and RBT-rand are less concentrated in Fig. 9c,d. RBT-det schedules overlap with peaks of traffic flow and crashes and the RBT-rand schedules are roughly evenly distributed. The locations of RBT-opt1, RBT-opt2, RBT-det, and RBT-rand test sites in Fig. 10 are represented by $\bigcirc$, $\Box$, $\times$, and $+$, respectively. In comparison with RBT-rand, the locations of RBT-opt1, RBT-opt2, and RBT-det concentrate to improve their performances. The test sites have different locations and rosters due to the different initial schedules in GA optimisation. The locations of RBT-det are concentrated around locations with either heavy traffic flow or many alcohol-related crashes.

**Figure 11:**
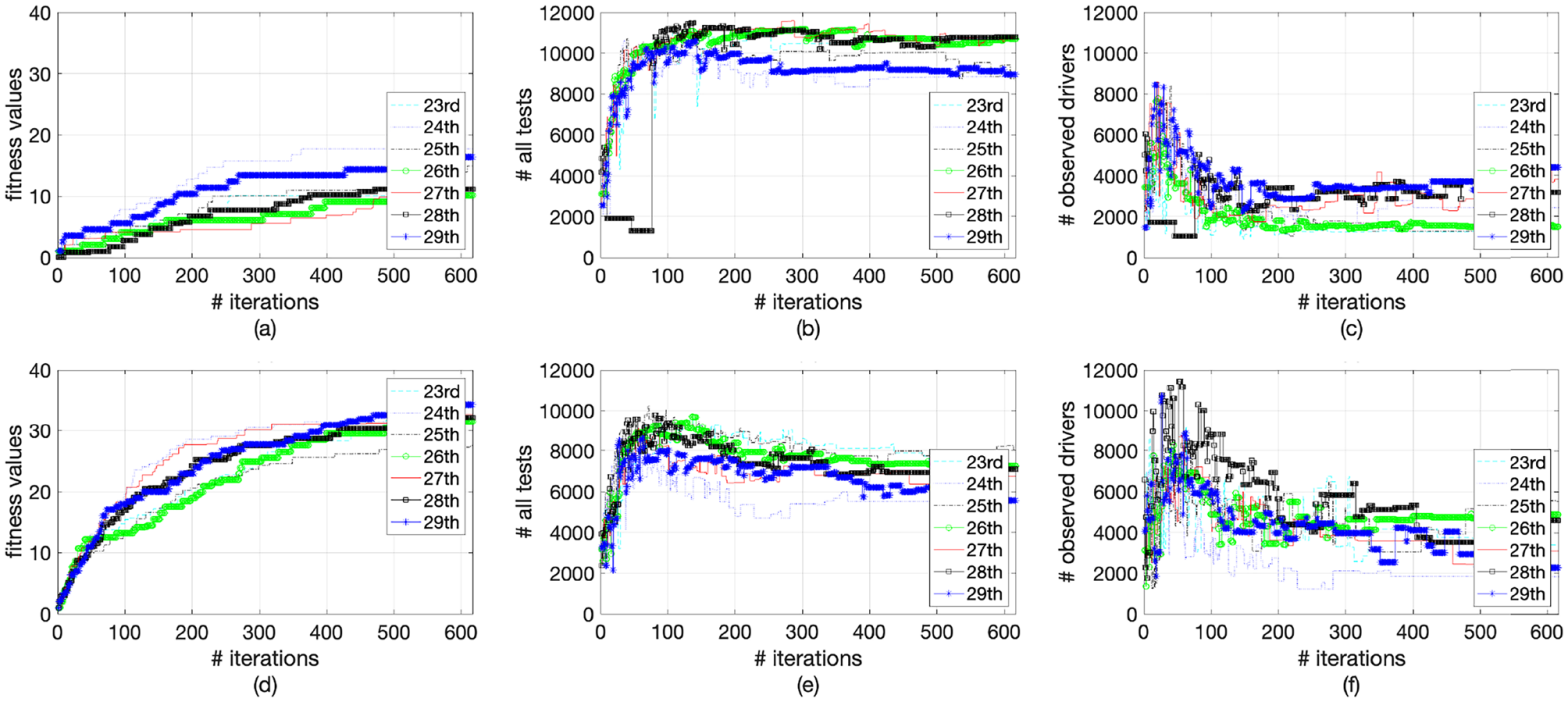
Fitness values, overall tests, and observing drivers for RBT-opt1 (**a**–**c**) and RBT-opt2 (**d**–**f**) schedules.

The values of the fitness function, total tests, and observing drivers for the considered period (23rd-29th) are given in Fig. 11a–f. The values are from the schedules with the maximal fitness values of each iteration. It can be seen in Fig. 11a,d that the maximal fitness values are non-decreasing. The curves of the number of all tests eventually gradually stabilize in Fig. 11b,e, and ultimately, there are more overall tests, compared with those at the start of the optimisation. It can be seen in Fig. 11b,e,c,f that there are more significant fluctuations on the curves of observing drivers than those of the total tests. This is because their coefficients are smaller and the curves are more vulnerable to variations.

The choice of weights in the fitness function (13) is a critical aspect of the optimization process, as it directly impacts the relative importance assigned to each of the three metrics in the fitness function: positive tests, total RBT conducted, and observing drivers (passersby). We acknowledge that the selection of weights can influence the optimization results.

Different values of $\alpha$, $\beta$, and $\gamma$ would normally lead to different optimal RBT schedules. It is possible to conduct more tests, achieve positive results, or increase the number of observing drivers by altering the values of $\alpha$, $\beta$, and $\gamma$. We have observed similar performance with minor parameter changes, but the details are not listed in the manuscript for the sake of brevity. Having said that, these parameters can provide levers for the policy maker and practitioner to steer the performance of the method towards their institutional aspirations. Note that with the current settings of the parameters, the optimal scheduling method can significantly increase the number of positive tests and also RBTs conducted with a minor decrease in the number of observing drivers.

## Limitations and future work

There are a number of limitations to this research.

- It is expected that the temporal and spatial coverage of traffic flow data is limited. To estimate the flows, a simple imputation method is used in Section "Traffic flow imputation". However, this method does not guarantee the computed traffic flow matrices are accurate. For example, the traffic flow along primary roads could be much higher than on secondary roads. Local traffic flows would fluctuate with different frequencies periodically. Such complexity requires in-depth modeling and estimation of traffic flows that take various factors into account. To utilize the proposed imputation approach in a wider range of situations, weight matrices can be introduced that take the fluctuations and correlations into account. Moreover, there are other methods to obtain more accurate estimation by integrating household travel surveys and sophisticated O-D estimation methods. These can be regarded as a future research direction.
- The limited resource for RBT schedules is modeled by (4a)–(4c). There are four key parameters, $N_{\max }$, $t_{\min }$, $t_{\max }$, and $\Delta$. On one hand, limited police force and budget may lead to finite values for $N_{\max }$ and $\Delta$ ($t_{\min }$ and $t_{\max }$ are less likely to vary over time), and higher values could easily help deliver a larger deterrence. On the other hand, further investigation is required to determine the point of diminishing return for increasing $N_{\max }$ and $\Delta$. Furthermore, there is significant randomness in traffic flow and unobservability in drink driving. Considering probabilistic attributes in (4b) and (4c) and devising advanced optimization methods with stochastic constraints are a future research direction to methodologically account for the intrinsic unobservability of the system.
- Victorian drink driving records are not available in this study. We used alcohol-related crashes instead as a proxy. However, they are not an ideal and complete substitute for drink driving records. The inherent insufficiency stems from sparse events that prevent any statistical inference. Integrating historical police checks (past RBT results) could to some extent alleviate this issue. Bayesian inference methods might be useful.
- The study utilizes data on alcohol-related crashes from the state of Victoria, Australia, but acknowledges limitations in Victoria’s reporting methodology. A crash might be categorised as involving alcohol if ‘suspected’ by the police, without direct Blood Alcohol Content (BAC) measurement, potentially leading to inaccuracies. While this does not significantly impact our findings, it is crucial to highlight it to avoid any misinterpretation of data and results.
- In Section "RBT performance results", random origins and destinations are used to simulate drink driving, and offenders are assumed to travel at 50km/h from the origins to the destinations. However, offenders may be more likely to appear in certain areas, and normally, the travel speeds are heavily influenced by the traffic conditions. By improving the assignment of origins and destinations (offender mapping), and adjusting the travel speeds according to the traffic conditions, we can obtain a more accurate picture of the underlying dynamics of drink driving. This is a research priority.
- RBT scheduling is a day-to-day process. Offenders might get accustomed to RBT test locations if they appear repetitively. In other words, RBT scheduling should consider inherent latent stochasticity in a day-to-day framework. This paper does not consider this and future research should address it.

Future research should investigate more accurate methods to estimate traffic flows and evaluate the accuracy in a convincing manner. Moreover, RBT scheduling can and perhaps should be considered jointly with drug testing to maximise general deterrence. Alcohol- and drug-related crashes can be used to obtain temporal and spatial distributions of the offense. Historical RBT results will help discover more insights into the behavioural patterns of the offenders. Coordinating with police force resource allocation can help evaluate the priorities and we will investigate how to use learning-based methods to resolve conflicts among different interests. It is crucial that testing schedules are compatible with existing task schedules. The RBT scheduling problem can be formulated as a (non-weighted) multi-objective optimisation problem. Future research should scrutinise this direction based on widely adopted Pareto front optimal solutions. More mathematically rigorous optimisation methods will help improve the quality of the solution. Finally, a more comprehensive procedure addressing all the above shortcomings is expected in the case of real-world implementation of optimisation of RBT schedules.

## Summary

This paper has studied the schedule optimisation problem for random breath tests (RBTs) at a large-scale network level to deliver general deterrence on drink driving. We present definitions of testing schedules, which are denoted by location, and start and finish times of testing. The limited police resources are described by inequalities, which include individual and total testing durations and the maximum number of test sites. The distribution of drink driving on any given date is obtained from the best match by clustering the historical crash data. We present a GA-based method to optimise roadside random breath test schedules. Real data of partial traffic flow measurements and alcohol-related crashes are used in the simulation tests for assessing the performance of testing schedules. The fitness values are calculated by the weighted sum of the RBT tests and the observing drivers. In the simulation runs, the optimised, deterministic, and random schedules are compared in terms of the numbers of RBT tests and observing drivers. The optimised schedules demonstrate significant improvements in detecting drink drivers, the number of overall tests, and the number of observing drivers, thus increasing the general deterrence.

## Data Availability

The datasets generated and analysed during the study are available from the corresponding author upon reasonable request.
